# T-cell Response Induced by the BNT162b2 COVID-19 Vaccine in Children

**DOI:** 10.1093/ofid/ofaf699

**Published:** 2025-11-20

**Authors:** Isabel Vogler, Stephanie Renken, Rolf Hilker, Bonny Gaby Lui, Andrew Finlayson, Alejandra Gurtman, Charulata Sabharwal, Nicholas Kitchin, Stephen Lockhart, William C Gruber, Kathrin U Jansen, Kena A Swanson, Özlem Türeci, Uğur Şahin

**Affiliations:** BioNTech SE, Mainz, Germany; BioNTech SE, Mainz, Germany; BioNTech SE, Mainz, Germany; BioNTech SE, Mainz, Germany; BioNTech SE, Mainz, Germany; Vaccine Research and Development, Pfizer Inc., Pearl River, New York, USA; Vaccine Research and Development, Pfizer Inc., Pearl River, New York, USA; Vaccine Research and Development, Pfizer Ltd., Marlow, UK; Vaccine Research and Development, Pfizer Ltd., Marlow, UK; Vaccine Research and Development, Pfizer Inc., Pearl River, New York, USA; Vaccine Research and Development, Pfizer Inc., Pearl River, New York, USA; Vaccine Research and Development, Pfizer Inc., Pearl River, New York, USA; BioNTech SE, Mainz, Germany; HI-TRON (Helmholtz Institute for Translational Oncology) Mainz by DKFZ, Mainz, Germany; BioNTech SE, Mainz, Germany; HI-TRON (Helmholtz Institute for Translational Oncology) Mainz by DKFZ, Mainz, Germany; TRON gGmbH-Translational Oncology, The University Medical Center of the Johannes Gutenberg University, Mainz, Germany

**Keywords:** COVID-19, SARS-CoV-2, vaccine, T-cell responses, children

## Abstract

Phase 1 and Phase 2/3 trials (NCT04816643) demonstrated that the mRNA-based coronavirus disease 2019 (COVID-19) vaccine BNT162b2 is tolerable and efficacious in (5–11-year-olds) following two 10 µg doses 21 days apart, and a third 10 µg dose after 6 months. Here, we show that vaccination induces neutralizing antibodies and T-cell immunogenicity in 10–11-year-old participants.

## MAIN

We have shown in adults that two 30 µg doses of the mRNA-based coronavirus disease 2019 (COVID-19) vaccine BNT162b2 (Comirnaty^®^, Pfizer/BioNTech COVID-19 vaccine) [1, 2] administered 21 days apart induced neutralizing antibodies as well as a polyepitopic T-cell response [3 , 4]. In adults, a third dose at least 6 months after the primary two-dose series effectively invigorated waning immune protection [5] by boosting severe acute respiratory syndrome coronavirus 2 (SARS-CoV-2) spike (S)-specific antibodies that cross-neutralized emerging variants of concern [6] (including Beta [B.1.351], Delta [B.1.617.2] and Omicron [B.1.1.529] variants). Our placebo-controlled Phase 2/3 randomized trial in children 5–11 years old demonstrated an acceptable safety profile, induction of neutralizing antibodies, and efficacy of a primary two-dose series of 10 µg BNT162b2 [7] against the original wild-type/Wuhan SARS-CoV-2 strain.

Here, we report follow-up data detailing 10 µg BNT162b2 vaccine-induced T-cell responses after the two-dose primary series and a third dose 6 months after dose 2 (D2), together with the effect of the third dose on cross-neutralizing antibodies in a subset of children 10–11 years old from the Phase 2/3 trial (BNT162b2: two doses; *n* = 22; three doses; *n* = 17; placebo: *n* = 12; Supplementary Tables 1 and 2, Supplementary Figure 1). This exploratory substudy was restricted to participants 10–11 years old, as the blood volumes required for immune cell analysis prohibited the inclusion of younger participants [8]. Blood samples were drawn on Day 1 (pre-dose 1, preD1), Day 29 (7 days post-dose 2, D2 + 7d), Day 203 (6 months post-dose 2, D2 + 6 m), Day 256 (pre-dose 3, preD3), and Day 288 (1-month post-dose 3, D3 + 1 m).

To characterize T-cell responses, we analyzed blood samples by interferon (IFN)γ enzyme-linked immunosorbent spot (ELISpot) and intracellular cytokine staining (ICS) (Supplementary Table 1, Supplementary Figure 1 for participant flow chart of the analysis set). Assays were performed following stimulation of peripheral blood mononuclear cells (PBMCs) with a peptide pool covering the N-terminal half of the wild-type S protein of SARS-CoV-2, with almost the entire S1 domain sequence, including the receptor binding domain (S pool 1). For later time points [9] the C-terminus with the entire S2 domain (S pool 2) containing the membrane fusion domain was included [10, 11]. The N-terminus shows less homology to other coronaviruses than the C-terminus, indicating that immune responses to N-terminus peptides would be more specific to SARS-CoV-2. Moreover, many SARS-CoV-2 variants have mutations within the N-terminus, making it an important region to target for broadly neutralizing antibody activity [12, 13].

**Figure 1.**
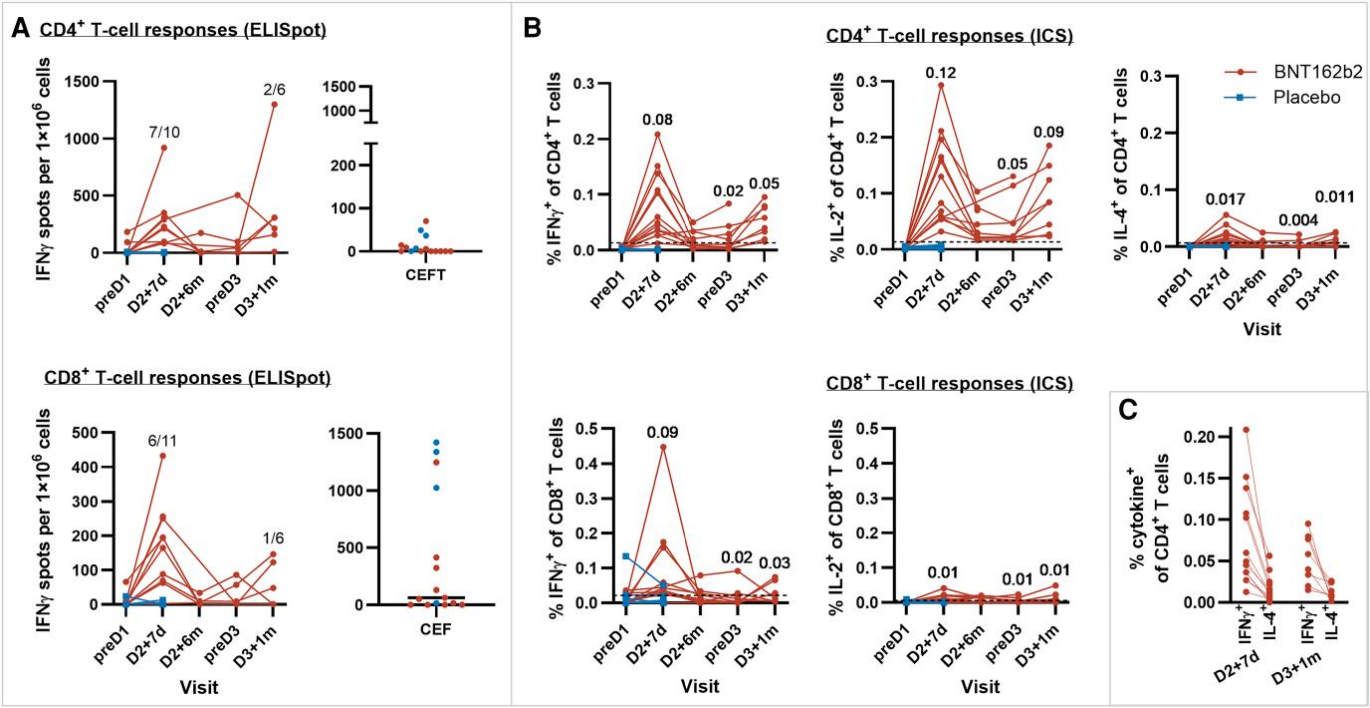
PBMCs were stimulated with Wuhan-Hu-1 spike protein-specific peptide pool 1 (S pool 1), CMV, EBV, influenza virus, and tetanus toxoid (CEFT), or CMV, EBV, and influenza virus (CEF) pools. (A) BNT162b2-induced CD4^+^ and CD8^+^ T-cell responses as assessed by IFNγ ELISpot. Circles represent background-corrected, normalized mean spot counts from duplicate wells for each participant. Values above the data points indicate the number of participants with detectable T-cell responses at D2 + 7d (responses compared to preD1, CD4^+^, n = 10 participants; CD8^+^, n = 11 participants) and D3 + 1 m (responses compared to preD3 except for one participant compared to D2 + 6 m due to a lack of data, CD4^+^ and CD8^+^ each n = 6 participants) relative to the total number of evaluable participants per cohort. Right panels include CD4^+^ (top) and CD8^+^ (bottom) T-cell responses to recall antigens CEFT and CEF, respectively, in all participants with a positive response at D2 + 7d. Horizontal bars indicate median values. Data are plotted for individual participants. (B) Cytokine polarization of BNT162b2-induced CD4^+^ and CD8^+^ T cells as assessed by flow cytometric ICS. S1-specific CD4^+^ or CD8^+^ T cells that produced the indicated cytokines are plotted as a proportion of total circulating T cells of the same subset. Values above the data points indicate mean percentages for BNT162b2-vaccinated participants at those time points. Dashed lines indicate limits of detection. Data from two participants who tested positive for SARS-CoV-2 were censored and not shown. (C) Comparison of the frequency of BNT162b2-induced S1-specific CD4^+^ T cells producing IFNγ versus IL-4 at D2 + 7d and D3 + 1 m. Data from two participants who tested positive for SARS-CoV-2 were censored and not shown. IFNγ spot-count data of the following participants were technically not evaluable and were not plotted: BNT162b2 group, n = 4 for CD4^+^ and n = 3 for CD8^+^; placebo group, n = 2 for CD4^+^ and for CD8^+^. Two participants who tested positive for SARS-CoV-2 at D2 + 6 m and two more at preD3 were censored from further ELISpot analysis. D, dose; d, day; m, month; IFN, interferon; IL, interleukin; CMV, Cytomegalovirus; EBV, Epstein–Barr virus; ICS, intracellular cytokine staining; ELISpot, enzyme-linked immunosorbent spot; PBMC, peripheral blood mononuclear cell.

IFNγ ELISpot was performed ex vivo without prior expansion, enabling detection of only high magnitude immune responses. SARS-CoV-2 S1-specific T-cell responses were not detectable at baseline by IFNγ ELISpot analysis. S1-specific CD4^+^ and CD8^+^ T cells were induced and detectable after the primary two-dose series of BNT162b2. At D2 + 7d, seven of ten (70.0%) and six of eleven (54.5%) participants had vaccine-induced de novo S1-specific CD4^+^ and CD8^+^ T-cell responses, respectively. Notably, S1-specific CD4^+^ T-cell responses were higher than, and CD8^+^ T-cell responses similar in magnitude to, the memory responses to common viruses and recall antigens (Figure 1*A*).

T-cell responses contracted following the two-dose series but were boosted by dose 3. At D3 + 1 m, as compared to preD3, two of six (33.3%) participants had vaccine-induced S1-specific CD4^+^ T-cell responses, and one of six (16.7%) participants showed CD8^+^ T-cell responses (Figure 1*A*). No participants who received a placebo exhibited S1-specific CD4^+^ or CD8^+^ T-cell responses.

ICS analysis, allowing for more sensitive detection of T-cell response magnitudes, revealed induction of S1-specific CD4^+^ T cells by D2 + 7d in almost all (fourteen of fifteen) BNT162b2-vaccinated participants, and not in participants who received placebo (zero of nine) (Figure 1*B*, Supplementary Table 1).

At D2 + 7d, 0.12% and 0.08% of vaccine-induced S1-specific CD4^+^ T cells were IL-2^+^ or IFNγ^+^, respectively. These increased frequencies were comparable to those reported in adults following the 30 µg booster dose [3] (0.11% for IL-2^+^ CD4^+^ and 0.07% for IFNγ^+^ CD4^+^ T cells) (Figure 1*B*). IL-4^+^ CD4^+^ T cells were rarely detected, indicating Th1 polarized CD4^+^ T-cell immunity (Figure 1*B*, *C*). By D2 + 6 m, S1-specific CD4^+^ T-cell responses remained detectable after contraction and were amplified by the third dose.

Production of IFNγ in S1-specific CD8^+^ T cells peaked at D2 + 7d (Figure 1*B*). One participant who received a placebo displayed preexisting S1-specific CD8^+^ T cells prior to vaccination on preD1, which declined by D2 + 7d, suggesting an undetected SARS-CoV-2 infection. By D2 + 6 m, S1-specific IFNγ^+^ CD8^+^ T cells decreased but remained above the limit of detection for two of eight participants. S1-specific IFNγ^+^ CD8^+^ T-cell responses increased by D3 + 1 m compared to D2 + 6 m. On D2 + 7d and D3 + 1 m, frequencies of S1-specific IL-2^+^ CD8^+^ T cells were low but detectable in nine of thirteen and six of eight participants, respectively.

S2-specific IFNγ^+^ CD4^+^ T-cell responses were detected by ELISpot in five of six (83.3%) participants by D3 + 1 m. S2-specific CD8^+^ T-cell responses were only detectable in one of six participants at D3 + 1 m (16.7%; Supplementary Figure 2a).

Production of IL-2 and IFNγ by S2-specific CD4^+^ T cells was higher at D3 + 1 m than D2 + 6 m and preD3 (Supplementary Figure 2b). When considering S1-specific responses, the CD4^+^ response to S2 indicates that BNT162b2 vaccination induced a polyepitopic T-cell response. Furthermore, the frequency of S2-specific CD8^+^ T cells was low or below the limit of detection following the third dose (Supplementary Figure 2b). Production of IL-4 by S2-specific T cells was low, again indicating a Th1 polarized T-cell response (Supplementary Figure 2c).

We then investigated the extent to which antibodies boosted by the third dose of BNT162b2 neutralize common Omicron subvariants of SARS-CoV-2 (BA.1, BA.2, BA.2.12.1, and BA.4/BA.5) using a vesicular stomatitis virus (VSV)-based SARS-CoV-2 pseudovirus neutralization (pVNT) assay (Supplementary Figure 3a, b). By D3 + 1 m, geometric mean neutralization titers (GMTs) against the Omicron subvariant pseudoviruses had increased 29.1- to 60.0-fold compared with D2 + 6 m. GMTs against wild-type pseudovirus increased by 19.2-fold on D3 + 1 m; at the same time point, GMTs against the Omicron subvariant pseudoviruses were 1.9- to 4.2-fold lower than GMTs against the wild-type pseudovirus.

Overall, our data demonstrate that two doses of 10 µg BNT162b2-induced de novo S-specific CD4^+^ T cells with a favorable Th1-skewed profile and functional S-specific CD8^+^ T-cell responses in children 10–11 years old. The magnitude of vaccine-induced CD8^+^ T-cell responses was detectable, albeit lower than reported in adults, which could be related to the lower vaccine dose, age-related differences in the immune responses, or other confounding factors in these small sample size cohorts. Recently, a study of children 5–11 years old reported 77.5% of participants who received two doses of 10 µg BNT162b2 (*n* = 40) developed SARS-CoV-2 S-specific T-cell responses within 2 weeks of the second vaccination using an assay set up that did not differentiate between CD4^+^ and CD8^+^ effector T cells [14].

We also show that a third dose of 10 µg BNT162b2, given over 6 months after the primary two-dose series, reinvigorated S-specific CD4^+^ T-cell responses and, to a lesser extent, CD8^+^ T-cell responses. A study of children in Singapore found no significant difference in T-cell responses at 12 months post-dose 3 between those who received only the primary two-dose series (*n* = 6) and those who received a third dose at 6 months (*n* = 9) [15]. The discrepancy with our results could be due to a lack of differentiation between CD4^+^ and CD8^+^ effector T cells, as well as a difference in follow-up times.

Finally, the third dose of 10 µg BNT162b2 (against the original wild-type/Wuhan SARS-Cov-2 strain) boosted relative antibody responses against Omicron BA.1, BA.2, and BA.4/BA.5 over those present 6 months after the primary two-dose series [14–16], and we demonstrated that this effect extends to Omicron BA.2.12.1.

The insights provided by this study are limited by the small sample size and no long-term follow-up of T-cell responses in participants who received the third dose. Due to the difference in follow-up intervals (1-week post-dose 2- and 1-month post-dose 3), the window of time for the peak of immune response post-dose 3 may not have been captured. Considering the smaller sample size post-dose 3 because of study withdrawal and breakthrough infections (Supplementary Figure 1), responses to doses 2 and 3 cannot be quantitatively compared. Furthermore, virus-neutralizing responses after dose 2 could not be assessed for all participants analyzed for cell-mediated immune responses due to the lack of paired serum samples. Data for children younger than 10 years old were not obtained because the blood volumes required for cellular analyses prohibited the inclusion of younger participants. This study enrolled healthy children; those with immune disorders/immunosuppression were not included, though they have been investigated elsewhere [17–19]. Additionally, since a modified 384-well ELISpot method adapted for smaller sample volumes from pediatric participants was used here, comparison with our published 96-well format ELISpot adult dataset is not possible [3]. Overall, because ELISpot assays were performed *ex vivo* without prior expansion, only immune responses of high magnitude could be detected.

Our findings show that, similar to responses in adults, a primary two-dose series of BNT162b2-induced robust T-cell responses in children and a third dose reinvigorates these responses and further boosts neutralizing antibody titers. A recently published study of a fourth dose utilizing a variant-adapted bivalent BNT162b2 (against original wild-type/Wuhan SARS-Cov-2 plus Omicron BA.4/BA.5) vaccination provided encouraging additional safety and reactogenicity data in the same population of 5- to 11-year-olds [20], which showed consistent trends to those reported here. Overall, these data support current vaccination strategies and the use of strain-adapted vaccines in pediatric populations.

## MATERIALS AND METHODS

### Blood/PBMC Samples

Blood samples for peripheral blood mononuclear cell (PBMC) collection were derived from 10- to 11-year-old children participating in the completed Phase 1/2/3 randomized trial (NCT04816643; first participant visit March 2021, last participant visit December 2023). Inclusion and exclusion criteria were previously reported [7]. Samples were collected on Day 1 (pre-dose 1, preD1), Day 29 (7 days post-dose 2, D2 + 7d), Day 203 (6 months post-dose 2, D2 + 6 m), Day 256 (pre-dose 3, preD3), and Day 288 (1 month post-dose 3, D3 + 1 m). Days represent the geometric mean of the range of sampling days for each time point post-dose 1. Participants were tested via PCR and SARS-CoV-2 N-binding antibody assay for breakthrough infections at the sampling time points. Exploratory objectives were to describe the cell-mediated immune response to vaccination with BNT162b2 and to assess the induction of cross-neutralizing serum activity against emerging SARS-CoV-2 variants. Consenting participants were recruited at sites selected for their ability to process PBMCs from children. PBMCs were isolated by Histopaque-1077 (Millipore-Sigma) density gradient centrifugation and cryopreserved prior to analysis. For full details regarding trial conduct and ethics, refer to the previous publications [7, 16].

### Peptides

Two pools of 15-mer peptides overlapping by 11 amino acids (aa) and covering the full sequence of the Wuhan-Hu-1 (wild-type) SARS-CoV-2 spike protein (GenBank: QHD43416.1) were used for *ex vivo* stimulation of PBMCs for flow cytometry and IFNγ enzyme-linked immunosorbent spot (ELISpot). S pool 1 covered the N-terminal portion [aa 1–643] that includes the receptor binding domain; S pool 2 covered the C-terminal part [aa 633–1,273]. Clinical-grade peptide pools were manufactured and supplied by JPT Peptide Technologies, GmbH, Berlin.

### Intracellular Cytokine Staining

Cytokine-producing T cells were quantified by intracellular cytokine staining (ICS). PBMCs were thawed and rested for 4 hours in OpTmizer medium supplemented with 2 µg/mL DNase I (Roche), then restimulated with wild-type SARS-CoV-2 spike protein represented by the peptide pools S pool 1 and 2 (2 µg/mL/peptide; JPT) in the presence of GolgiPlug (BD) for 18 hours at 37°C. Controls were treated with dimethyl sulfoxide (DMSO)-containing medium. Positive controls were stimulated with anti-CD3 antibody (1:1000) and CEFX (containing peptide epitopes from Clostridium tetani, Coxsackievirus B4, Haemophilus influenza, Helicobacter pylori, Human adenovirus 5, Human herpesvirus 1, Human herpesvirus 2, Human herpesvirus 3, Human herpesvirus 4, Human herpesvirus 5, Human herpesvirus 6, Human papillomavirus, Influenza A, JC polyomavirus, Measles virus, Rubella virus, Toxoplasma gondii, Vaccinia virus) peptide mix. Cells were stained for viability (Fixable Viability Dye eFluor™ 780, 1:100; eBioscience) and surface markers (CD3 BV421 [clone UCHT1], 1:250; CD4 BV480 [clone RPA-T4], 1:50; CD8 BB515 [clone RPA-T8], 1:100; all BD Biosciences) in Brilliant Stain Buffer (BD Biosciences) for 20 minutes at 4°C. Samples were fixed and permeabilized using the Cytofix/Cytoperm kit according to manufacturer's instructions (BD Biosciences). Intracellular staining (CD3 BV421, 1:250; CD4 BV480, 1:50; CD8 BB515, 1:100; IFNγ BB700 [clone B27], 1:250; IL-2 PE [clone MQ1-17H12], 1:10; IL-4 APC [clone MP4-25D2], 1:500; all BD Biosciences) was performed in Perm/Wash buffer supplemented with Brilliant Stain Buffer Plus for 30 minutes at 4°C. Samples were acquired on a fluorescence-activated cell sorter (FACS) LYRIC instrument (BD Biosciences) and analyzed with FlowJo software version 10.7.1 (FlowJo LLC, BD Biosciences). Cytokine production was corrected for background by subtraction of values obtained with DMSO-containing medium. Representative raw, background-unsubtracted frequencies of IFNγ-specific T-cell responses are reported in Supplementary Tables 3 and 4. The lower limit of detection was determined using the specific peptide pools. Negative values were set to zero.

### IFNγ ELISpot

IFNγ ELISpot analysis was performed *ex vivo* without prior cell expansion, using PBMCs depleted of CD4^+^ and enriched for CD8^+^ T cells (yielding “CD8^+^ effectors”) or depleted of CD8^+^ and enriched for CD4^+^ T cells (yielding “CD4^+^ effectors”). ELISpot plates (384 well, CTL ImmunoSpot) coated with IFNγ-specific capture antibodies [1:250; CTL], were washed with phosphate-buffered saline (PBS) and blocked with X-VIVO 15 medium (Lonza) containing 2% human serum albumin (CSL-Behring) for 2–4 hours. Per well, 1.1 × 10^5^ enriched effector cells were stimulated with wild-type SARS-CoV-2 spike protein represented by peptide pools S pool 1 and 2 (2 µg/mL/peptide) in technical duplicate. A further single sample was stimulated with positive control anti-CD3 monoclonal antibody (CD3-2 [1:1,000; Mabtech]) to facilitate normalization to T-cell fitness and sample quality. CEF and CEFT (containing peptides from Infectious mononucleosis, Cytomegalovirus disease, Influenza, Flu-like disease and Tetanus, Infectious mononucleosis, Cytomegalovirus disease, Influenza, and Flu-like disease, respectively) pools were additionally used in stimulations as positive controls. After incubation for 18 hours, spots were visualized using a biotinylated anti-human IFNγ detection antibody followed by incubation with a streptavidin-labelled alkaline phosphatase and 5-bromo-4-chloro-3′-indolyl phosphate (BCIP)/nitro blue tetrazolium (NBT) substrate (ELISpot Hu IFNγ BCIP SCE Kit, CTL ImmunoSpot). Plates were scanned using a Robot ELISpot Reader and analyzed using ImmunoCapture V7.0.7.0 software. Spot counts were displayed as mean values of each duplicate. IFNγ secretion by T cells induced by antigen-specific peptide stimulation was compared with stimulation with DMSO-only media as a negative control using an in-house ELISpot data analysis tool (EDA), based on two statistical tests (distribution-free resampling) according to Moodie et al. [21, 22], to provide sensitivity while maintaining control over false positives [21, 22].

Spot-count values from the stimulation with anti-CD3 antibody were used to normalize the IFNγ secretion values, to account for varying sample quality (which also served as a positive control for cell functionality). Normalization to facilitate direct comparison of spot counts and strength of response between individuals was performed as follows. The dependency was modeled in a log-linear fashion using a Bayesian model including a noise component (unpublished). Each normalization was sampled 10,000 times from the model, and the median was taken as the normalized spot-count value. Likelihood of the model: $\log\;\lambda_{E}=\alpha \log\;\lambda_{P}+\log\;\beta_{j}+\sigma \varepsilon$, where $\;\lambda_{E}$ is the normalized spot-count of the sample; *α* is a stable factor (normally distributed) common among all positive controls $\lambda_{P}$; $\beta_{j}$ is a sample *j*-specific component (normally distributed); and σε is the noise component, of which *σ* is Cauchy distributed, and ε is Student's *t* distributed. $\beta_{j}$ ensures that each sample is treated as a different batch.

### Vesicular Stomatitis Virus (VSV) SARS-CoV-2 S Variant Pseudovirus Generation

A recombinant replication-deficient VSV vector that encoded green fluorescent protein (GFP) and luciferase instead of the VSV-glycoprotein (VSV-G) was pseudotyped with SARS-CoV-2 S glycoprotein derived from either the wild-type reference strain (NCBI Ref: 43740568) or the Omicron BA.1 variant (GISAID: EPI_ISL_6590782; alterations: A67V, Δ69/70, T95I, G142D, Δ143-145, Δ211, L212I, ins214EPE, G339D, S371L, S373P, S375F, K417N, N440K, G446S, S477N, T478K, E484A, Q493R, G496S, Q498R, N501Y, Y505H, T547K, D614G, H655Y, N679K, P681H, N764K, D796Y, N856K, Q954H, N969K, and L981F), Omicron BA.2 (GISAID: EPI_ISL_6795834; alterations: T191I, Δ24-26, A27S, G142D, V213G, G339D, S371F, S373P, S375F, T376A, D405N, R408S, K417N, N440K, S477N, T478K, E484A, Q493R, Q498R, N501Y, Y505H, D614G, H655Y, N679K, P681H, N764K, D796Y, Q954H, and N969K), Omicron BA.4/5 (GISAID: EPI_ISL_11017528; alterations: T191I, Δ24-26, A27S, Δ69/70, G142D, V213G, G339D, S371F, S373P, S375F, T376A, D405N, R408S, K417N, N440K, L452R, S477N, T478K, E484A, F486V, Q498R, N501Y, Y505H, D614G, H655Y, N679K, P681H, N764K, D796Y, Q954H, and N969K), and Omicron BA.2.12.1 (GISAID: EPI_ISL_9767878; alterations: T191I, Δ24-26, A27S, G142D, V213G, G339D, S371F, S373P, S375F, T376A, D405N, R408S, K417N, N440K, L452Q, S477N, T478K, E484A, Q493R, Q498R, N501Y, Y505H, D614G, H655Y, N679K, P681H, S704L, N764K, D796Y, Q954H, and N969K) variants according to published pseudotyping protocols [23].

In brief, HEK293T/17 monolayers (ATCC^®^ CRL-11268™) cultured in Dulbecco's modified Eagle's medium (DMEM) with GlutaMAX™ (Gibco) supplemented with 10% heat-inactivated fetal bovine serum (FBS [Sigma-Aldrich]) (referred to as medium) were transfected with a Sanger sequencing-verified variant-specific SARS-CoV-2 S expression plasmid with Lipofectamine LTX (Life Technologies) following the manufacturer's instructions. At 24 hours after transfection, the cells were infected at a multiplicity of infection (MOI) of three with VSV-G-complemented VSVΔG vector. After incubation for 2 hours at 37°C with 7.5% CO_2_, cells were washed twice with PBS before medium supplemented with anti-VSV-G antibody (clone 8G5F11, Kerafast Inc.) was added to neutralize residual VSV-G-complemented input virus. VSV-SARS-CoV-2-S pseudotype-containing medium was harvested 20 hours after inoculation, passed through a 0.2-µm filter (Nalgene) and stored at –80°C. The pseudovirus batches were titrated on Vero 76 cells (ATCC^®^ CRL-1587™) cultured in medium. The relative luciferase units induced by a defined volume of a SARS-CoV-2 wild-type strain S glycoprotein pseudovirus reference batch, which corresponds to an infectious titer of 200 transducing units (TU) per mL, was used as a comparator; the reference batch has been previously described [24]. Input volumes for the SARS-CoV-2 variant pseudovirus batches were calculated to normalize the infectious titer based on the relative luciferase units relative to the reference.

### Pseudovirus Neutralization Assay

Vero 76 cells were seeded in 96-well white, flat-bottom plates (Thermo Scientific) at 40,000 cells/well in medium 4 hours prior to the assay and cultured at 37°C with 7.5% CO_2_. Each individual serum was serially diluted 2-fold in medium, with the first dilution being 1:5. VSV-SARS-CoV-2-S particles were diluted in medium to obtain 200 TU in the assay. Serum dilutions were mixed 1:1 with pseudovirus (*n* = 2 technical replicates per serum per pseudovirus) for 30 minutes at room temperature before being added to Vero 76 cell monolayers and incubated at 37°C with 7.5% CO_2_ for 24 hours. Supernatants were removed, and the cells were lysed with luciferase reagent (Promega). Luminescence was recorded on a CLARIOstar^®^ Plus microplate reader (BMG Labtech), and neutralization titers were calculated as the reciprocal of the highest serum dilution that still resulted in a 50% reduction in luminescence. Results for all pseudovirus neutralization experiments were expressed as geometric mean titers (GMT) of duplicates. If no neutralization was observed, an arbitrary titer value of half of the limit of detection (LOD) was reported.

### Data and Statistical Analyses

Demographics for the subset of the pediatric population analyzed for cell-mediated immune responses are shown in Supplementary Table 2. Data were plotted using GraphPad Prism software 9.1.0.

## Supplementary Material

ofaf699_Supplementary_Data

## Supplementary Data

Supplementary materials are available at *Open Forum Infectious Diseases* online. Consisting of data provided by the authors to benefit the reader, the posted materials are not copyedited and are the sole responsibility of the authors, so questions or comments should be addressed to the corresponding author.
