# Supplementary material for: T-cell Response Induced by the BNT162b2 COVID-19 Vaccine in Children
Source: Open Forum Infect Dis. 2025 Nov 20;12(12):ofaf699. doi: 10.1093/ofid/ofaf699 (PMC12680099; doi:10.1093/ofid/ofaf699)
Supplement: ofaf699_Supplementary_Data [file ofaf699_supplementary_data.docx]

**Supplementary figures and tables**

**Participant disposition**

**Supplemental Table 1. Analysis sets**

| **Study treatment** | **Vaccination** | | | **ICS analysis** | | | | | **ELISpot analysis** | | | | | **pVNT analysis** | |
| --- | --- | --- | --- | --- | --- | --- | --- | --- | --- | --- | --- | --- | --- | --- | --- |
|  | **Dose 1 (D1)** | **Dose 2 (D2)** | **Dose 3 (D3)** | **preD1** | **D2+7d** | **D2+6m** | **preD3** | **D3+1m** | **preD1** | **D2+7d** | **D2+6m** | **preD3** | **D3+1m** | **D2+6m** | **D3+1m** |
| 10 µg BNT162b2 | 22 | 22 | 17 | 15 [8] | 15 [8] | 13 [3] | 10 [3] | 9  [3] | 15  [8] | 15 [8] | 9  [3] | 9  [3] | 10  [3] | 17 | 17 |
| Placebo | 12 | 12 |  | 9 [3] | 9 [3] |  |  |  | 6 [3] | 6  [3] |  |  |  |  |  |

Values indicate number of participants for whom ICS, ELISpot, or pVNT analysis was performed. Numbers in square brackets refer to the extent of overlap between the ICS and ELISpot assays. Empty boxes indicate no participants in the placebo group received a placebo dose or were analyzed at those time points. ICS = intracellular cytokine staining; ELISpot = enzyme-linked immunosorbent spot; pVNT = pseudovirus neutralization titer.

**
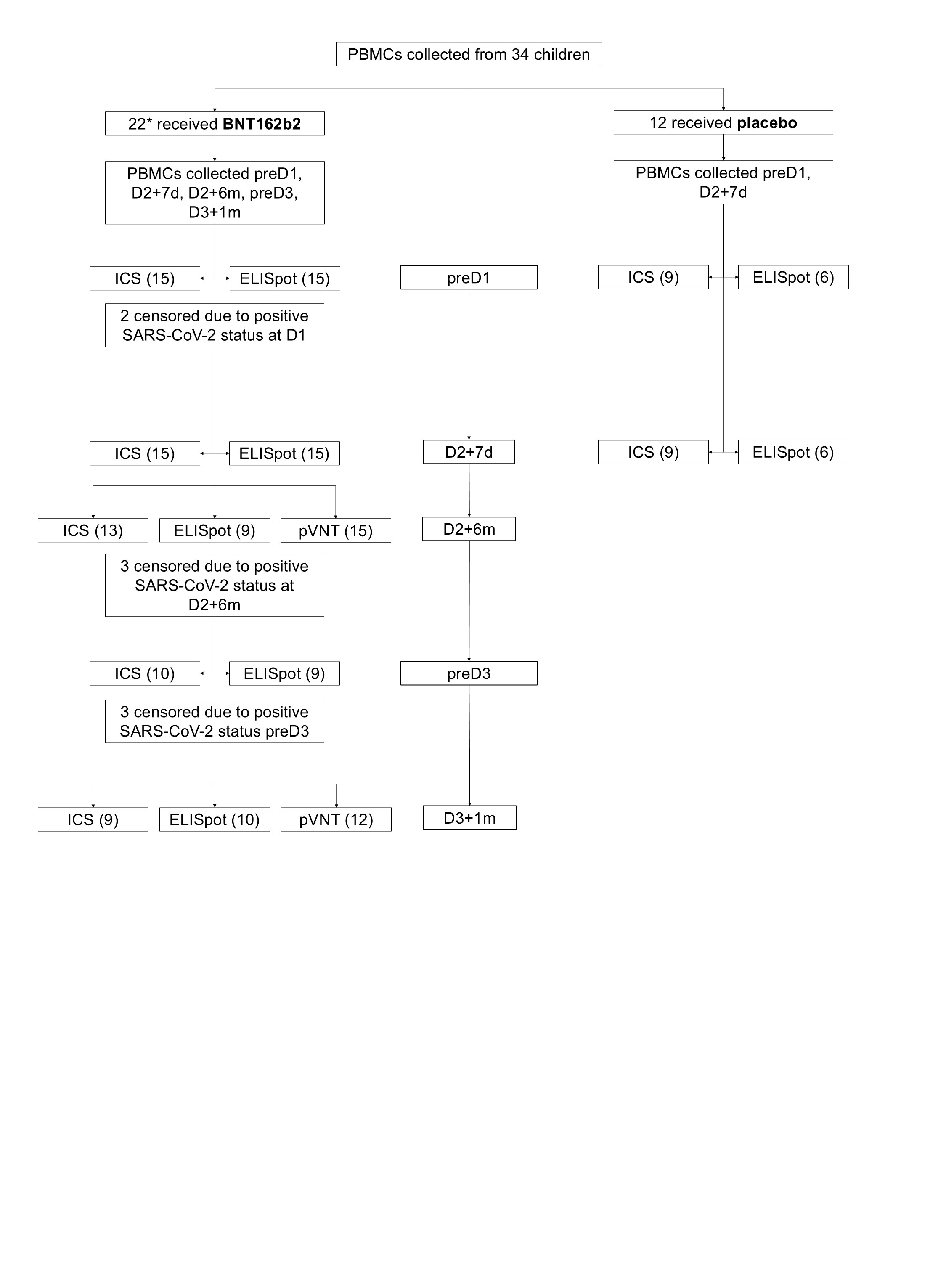
**

**Supplemental Figure 1. Flow chart showing the analysis sets of 10- to 11-year-old participants**

Parentheses indicate number of participants analyzed per analysis set prior to censors. *Twenty-two participants received 10 µg BNT162b2 at dose 1 and 2, of which 17 participants received a third dose. Participants positive for SARS-CoV-2 were censored at the time of positive status and in all follow-up analysis sets. ICS = intracellular cytokine staining; ELISpot = enzyme-linked immunosorbent spot; pVNT = pseudovirus neutralization titer.

**Supplemental Table 2. Demographics and baseline characteristics for all participants analyzed at start of study enrollment**

| **Group** | **BNT162b2 (N=22)** | **Placebo (N=12)** | **Total (N=34)** |
| --- | --- | --- | --- |
| **Sex, n (%)** |  |  |  |
| Male | 11 (50.0%) | 7 (58.3%) | 18 (52.9%) |
| Female | 11 (50.0%) | 5 (41.7%) | 16 (47.1%) |
| **Race, n (%)** |  |  |  |
| White | 18 (81.8%) | 8 (66.7%) | 26 (76.5%) |
| Black or African   American | 4 (18.2%) | 2 (16.7%) | 6 (17.6%) |
| American Indian or   Alaska Native | 0 | 1 (8.3%) | 1 (2.9%) |
| Asian | 0 | 1 (8.3%) | 1 (2.9%) |
| Native Hawaiian or   other Pacific Islander | 0 | 0 | 0 |
| Multiple | 0 | 0 | 0 |
| Not Reported | 0 | 0 | 0 |
| **Ethnicity, n (%)** |  |  |  |
| Hispanic or Latino | 1 (4.5%) | 0 | 1 (2.9%) |
| Not Hispanic or Latino | 21 (95.5%) | 12 (100.0%) | 33 (97.1%) |
| Not Reported | 0 | 0 | 0 |
| **Age (years)** |  |  |  |
| Mean (SD) | 10.23 (0.429) | 10.42 (0.515) | 10.29 (0.462) |
| Median (Range) | 10.00 (10.0, 11.0) | 10.00 (10.0, 11.0) | 10.00 (10.0, 11.0) |
| **Obese^*^** |  |  |  |
| Yes | 7 (31.8%) | 2 (16.7%) | 9 (26.5%) |
| No | 15 (68.2%) | 10 (83.3%) | 25 (73.5%) |
| **Baseline SARS-CoV-2 Status** |  |  |  |
| Positive | 2 (9.1%) | 0 | 2 (5.9%) |
| Negative | 20 (90.9%) | 12 (100.0%) | 32 (94.1%) |
| **Coexisting conditions^†^** |  |  |  |
| Yes | 7 (31.8%) | 3 (25.0%) | 10 (29.4%) |
| No | 15 (68.2%) | 9 (75.0%) | 24 (70.6%) |

^*^ Obese was defined as a body mass index (BMI) at or above the 95^th^ percentile according to the growth chart. Refer to the CDC growth charts at <https://www.cdc.gov/growthcharts/cdc-charts.htm>

^†^ Coexisting conditions were defined as those that increase the risk of severe Covid-19 (i.e., one or more prespecified underlying conditions as defined in Kim et al. ^25^, obesity, or both

**
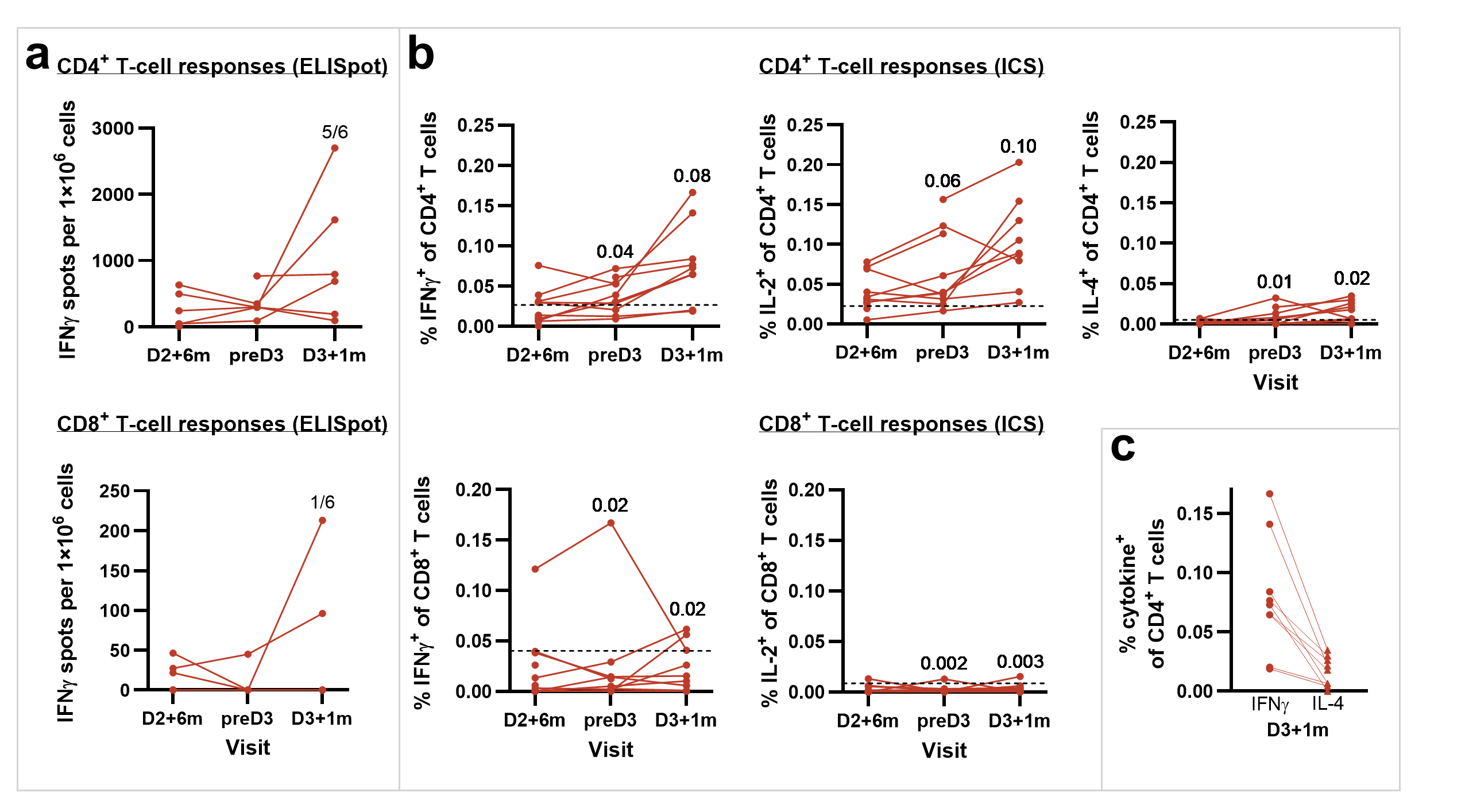
**

**Supplemental Figure 2. A third dose of BNT162b2 boosts CD4^+^ and CD8^+^ T-cell responses to S pool 2 in experienced 10- to 11-year-old children**

Peripheral blood mononuclear cells (PBMCs) obtained on day 203 (6 months post-dose 2, D2+6m), day 256 (pre-dose 3, preD3), and day 288 (1 month post-dose 3, D3+1m) were stimulated with Wuhan-Hu-1 spike protein-specific peptide pool 2 (S pool 2). (**a**) BNT162b2-boosted IFNγ^+^ CD4^+^ and CD8^+^ T-cell responses as assessed by IFNγ ELISpot analysis. PBMCs were enriched for CD4^+^ or CD8^+^ T-cell effectors and stimulated overnight with S pool 2. Circles represent the background corrected, normalized mean spot count from duplicate wells for each study participant. Values above the data points indicate the number of participants with a detectable T-cell response at D3+1m relative to the total number of evaluable participants per cohort. (**b**) Cytokine polarization of BNT162b2-boosted CD4^+^ and CD8^+^ T cells as assessed by flow cytometric intracellular cytokine staining (ICS). S pool 2-specific CD4^+^ or CD8^+^ T cells that produced the indicated cytokines are plotted as a proportion of total circulating T cells of the same subset. Values above the data points indicate the mean of percentages per treatment group at those time points. Dashed lines indicate the limits of detection. Data are plotted from individual participants. (**c**) Comparison of the frequency of BNT162b2-boosted S pool 2-specific CD4^+^ T cells producing IFNγ^+^ versus IL-4^+^ at D3+1m. Data from two study participants who tested positive for SARS-CoV-2 at the first vaccination have been censored and are not shown. Two participants who tested positive for SARS-CoV-2 at D2+6m and two more at preD3 were censored from further analysis within the ELISpot data.

**
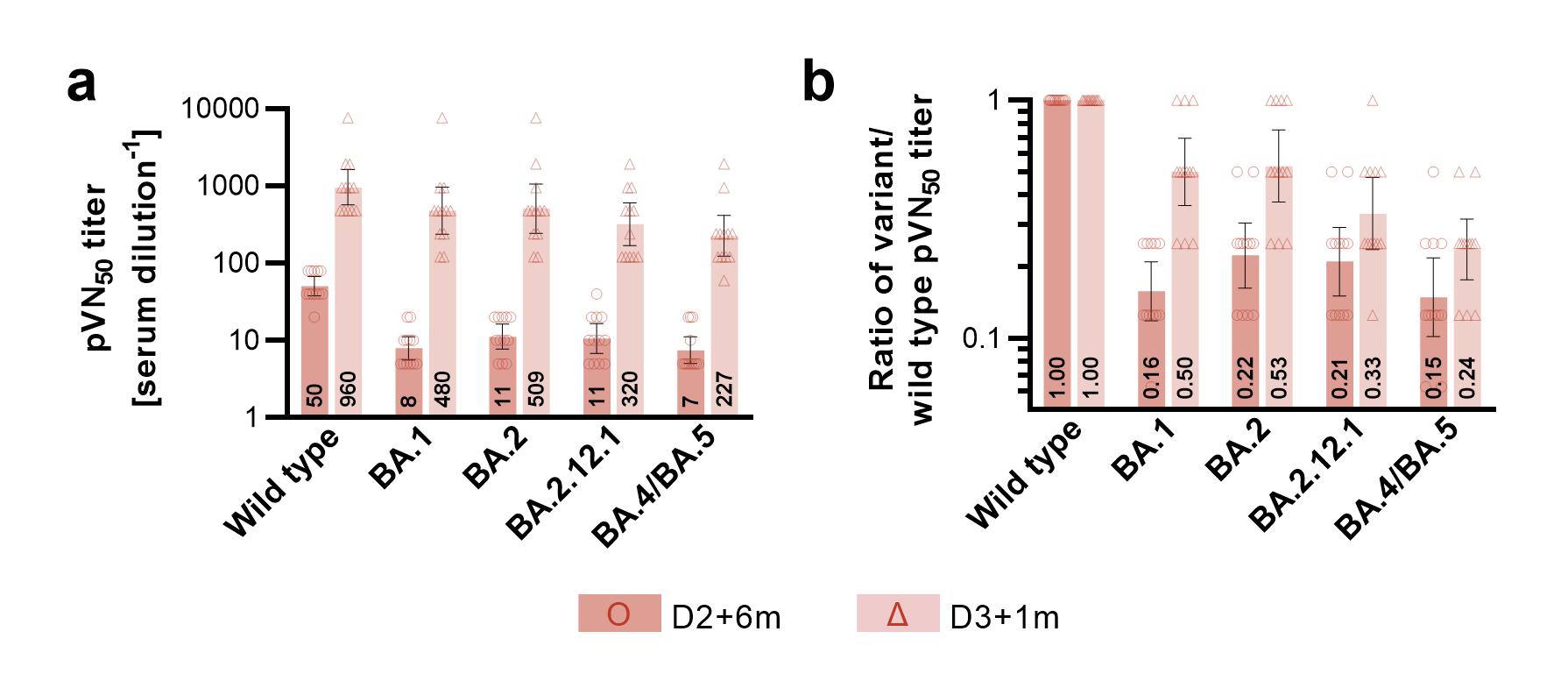
**

**Supplemental Figure 3. A third dose of BNT162b2 boosts virus neutralization titers against Omicron variants.**

Participant sera collected on day 256 (pre-dose 3, preD3) and day 288 (1 month post-dose 3, D3+1m) was exposed to pseudoviruses displaying variants of SARS-CoV-2, including wild-type and the Omicron variants BA.1, BA.2, BA.2.12.1, and BA.4/BA.5. (**a**) Pseudovirus 50% neutralization titers (pVNT_50_) expressed as the mean of duplicate titers. (**b**) Ratios of variant titers to wild-type titers with wild-type values set to 1. Data from one study participant who tested positive for SARS-CoV-2 at the first vaccination have been censored and are not shown. One participant who tested positive for SARS-CoV-2 at D2+6m and three more at preD3 were additionally censored from pVNT analysis.

**Supplemental Table 3. Raw frequencies of IFN**γ**-producing CD4^+^ T cells in participants**

| **Participant group** | **Visit** | **Non-stimulated*** | **S1-stimulated** |
| --- | --- | --- | --- |
| **BNT162b2** | **preD1** | **0.007586** | **0.01067** |
|  | **D2+7d** | **0.006917** | **0.066809** |
|  | **D2+6m** | **0.02059** | **0.029816** |
|  | **preD3** | **0.014734** | **0.020505** |
|  | **D3+1m** | **0.017387** | **0.057348** |
| **BNT162b2** | **preD1** | **0.007323** | **0.01168** |
|  | **D2+7d** | **0.008151** | **0.057667** |
|  | **D2+6m** | **0.022231** | **0.023988** |
|  | **preD3** | **0.006845** | **0.010967** |
|  | **D3+1m** | **0.015688** | **0.095305** |
| **BNT162b2** | **preD1** | **0.005658** | **0.008289** |
|  | **D2+7d** | **0.014914** | **0.027552** |
|  | **D2+6m** | **0.008885** | **0.017136** |
|  | **preD3** | **0.00406** | **0.007635** |
|  | **D3+1m** | **0.005229** | **0.020175** |
| **BNT162b2** | **preD1** | **0.006393** | **0.014548** |
|  | **D2+7d** | **0.016435** | **0.052666** |
|  | **D2+6m** | **0.006431** | **0.026811** |
|  | **preD3** | **0.010944** | **0.040312** |
|  | **D3+1m** | **0.010672** | **0.087171** |
| **BNT162b2** | **preD1** | **0.010183** | **0.004001** |
|  | **D2+7d** | **0.016764** | **0.061591** |
| **BNT162b2** | **preD1** | **0.012268** | **0.015169** |
|  | **D2+7d** | **0.019754** | **0.055643** |
|  | **D2+6m** | **0.011542** | **0.021928** |
|  | **preD3** | **0.003827** | **0.014579** |
|  | **D3+1m** | **0.008265** | **0.041555** |
| **BNT162b2** | **preD1** | **0.013148** | **0.015987** |
|  | **D2+7d** | **0.013232** | **0.165046** |
|  | **D2+6m** | **0.010436** | **0.02576** |
| **BNT162b2** | **preD1** | **0.010809** | **0.013746** |
|  | **D2+7d** | **0.018151** | **0.044815** |
|  | **preD3** | **0.009744** | **0.053058** |
|  | **D3+1m** | **0.010375** | **0.068576** |
| **BNT162b2** | **preD1** | **0.009706** | **0.008742** |
|  | **D2+7d** | **0.008454** | **0.035295** |
|  | **D2+6m** | **0.003907** | **0.00862** |
|  | **preD3** | **0.006812** | **0.008886** |
|  | **D3+1m** | **0.004437** | **0.024003** |
| **BNT162b2** | **preD1** | **0.013123** | **0.016369** |
|  | **D2+7d** | **0.020002** | **0.158475** |
|  | **D2+6m** | **0.006748** | **0.057074** |
|  | **preD3** | **0.003933** | **0.087521** |
| **BNT162b2** | **preD1** | **0.010417** | **0.01493** |
|  | **D2+7d** | **0.011342** | **0.090499** |
| **BNT162b2** | **preD1** | **0.015806** | **0.01523** |
|  | **D2+7d** | **0.013894** | **0.116363** |
|  | **D2+6m** | **0.01017** | **0.017746** |
| **BNT162b2** | **preD1** | **0.018312** | **0.0104** |
|  | **D2+7d** | **0.015729** | **0.123467** |
|  | **D2+6m** | **0.009079** | **0.014426** |
| **BNT162b2** | **preD1** | **0.009469** | **0.010539** |
|  | **D2+7d** | **0.006056** | **0.21484** |
|  | **D2+6m** | **0.005644** | **0.039225** |
|  | **preD3** | **0.002327** | **0.02142** |
|  | **D3+1m** | **0.003141** | **0.098514** |
| **Placebo** | **preD1** | **0.012302** | **0.003806** |
|  | **D2+7d** | **0.008944** | **0.008901** |
| **Placebo** | **preD1** | **0.012965** | **0.005565** |
|  | **D2+7d** | **0.017294** | **0.014377** |
| **Placebo** | **preD1** | **0.006614** | **0.003231** |
|  | **D2+7d** | **0.007562** | **0.009371** |
| **Placebo** | **preD1** | **0.006186** | **0.010173** |
|  | **D2+7d** | **0.020971** | **0.015788** |
| **Placebo** | **preD1** | **0.006988** | **0.007434** |
|  | **D2+7d** | **0.012504** | **0.008691** |
| **Placebo** | **preD1** | **0.004847** | **0.008488** |
|  | **D2+7d** | **0.008677** | **0.007656** |
| **Placebo** | **preD1** | **0.010286** | **0.012339** |
|  | **D2+7d** | **0.016943** | **0.015317** |
| **Placebo** | **preD1** | **0.011969** | **0.011864** |
|  | **D2+7d** | **0.018708** | **0.012874** |
| **Placebo** | **preD1** | **0.010973** | **0.005147** |
|  | **D2+7d** | **0.014043** | **0.012695** |

*Non-stimulated refers to DMSO controls

**Supplemental Table 4. Raw frequencies of IFN**γ**-producing CD8^+^ T cells in participants**

| **Participant group** | **Visit** | **Non-stimulated*** | **S1-stimulated** |
| --- | --- | --- | --- |
| **BNT162b2** | **preD1** | **0.092397** | **0.1284** |
|  | **D2+7d** | **0.115251** | **0.160241** |
|  | **D2+6m** | **0.109835** | **0.188583** |
|  | **preD3** | **0.097305** | **0.188915** |
|  | **D3+1m** | **0.058462** | **0.072197** |
| **BNT162b2** | **preD1** | **0.015845** | **0.011231** |
|  | **D2+7d** | **0.009438** | **0.044903** |
|  | **D2+6m** | **0.045078** | **0.051139** |
|  | **preD3** | **0.015** | **0.014704** |
|  | **D3+1m** | **0.007063** | **0.03448** |
| **BNT162b2** | **preD1** | **0.012599** | **0.041051** |
|  | **D2+7d** | **0.021759** | **0.053057** |
|  | **D2+6m** | **0.034907** | **0.029981** |
|  | **preD3** | **0.021361** | **0.023414** |
|  | **D3+1m** | **0.027932** | **0.101553** |
| **BNT162b2** | **preD1** | **0.004053** | **0.006886** |
|  | **D2+7d** | **0.013469** | **0.042202** |
|  | **D2+6m** | **0.012615** | **0.024174** |
|  | **preD3** | **0.01317** | **0.009878** |
|  | **D3+1m** | **0.010882** | **0.039037** |
| **BNT162b2** | **preD1** | **0.034633** | **0.03586** |
|  | **D2+7d** | **0.022289** | **0.063254** |
| **BNT162b2** | **preD1** | **0.026951** | **0.019727** |
|  | **D2+7d** | **0.00775** | **0.037783** |
|  | **D2+6m** | **0.035491** | **0.032898** |
|  | **preD3** | **0.007658** | **0.035581** |
|  | **D3+1m** | **0.020381** | **0.025203** |
| **BNT162b2** | **preD1** | **0.020037** | **0.01721** |
|  | **D2+7d** | **0.005459** | **0.179235** |
|  | **D2+6m** | **0.01534** | **0.029059** |
| **BNT162b2** | **preD1** | **0.016595** | **0.009088** |
|  | **D2+7d** | **0.017127** | **0.060026** |
|  | **preD3** | **0.003988** | **0.016276** |
|  | **D3+1m** | **0.005827** | **0.009997** |
| **BNT162b2** | **preD1** | **0.011703** | **0.021669** |
|  | **D2+7d** | **0.068668** | **0.516035** |
|  | **D2+6m** | **0.03706** | **0.01564** |
|  | **preD3** | **0.022597** | **0.028857** |
|  | **D3+1m** | **0.024126** | **0.030337** |
| **BNT162b2** | **preD1** | **0.026183** | **0.037167** |
|  | **D2+7d** | **0.01383** | **0.17235** |
|  | **D2+6m** | **0** | **0.033285** |
|  | **preD3** | **0.009757** | **0.030453** |
| **BNT162b2** | **preD1** | **0.046891** | **0.078409** |
|  | **D2+7d** | **0.034442** | **0.04634** |
| **BNT162b2** | **preD1** | **0.014801** | **0.015528** |
|  | **D2+7d** | **0.022408** | **0.016517** |
|  | **D2+6m** | **0.031916** | **0.019277** |
| **BNT162b2** | **preD1** | **0.017678** | **0.024409** |
|  | **D2+7d** | **0.017343** | **0.041449** |
|  | **D2+6m** | **0.008611** | **0.015388** |
| **BNT162b2** | **preD1** | **0.016628** | **0.011749** |
|  | **D2+7d** | **0.018077** | **0.076653** |
|  | **D2+6m** | **0.008681** | **0.031367** |
|  | **preD3** | **0.028523** | **0.031836** |
|  | **D3+1m** | **0.019971** | **0.085292** |
| **Placebo** | **preD1** | **0.02335** | **0.046526** |
|  | **D2+7d** | **0.0106** | **0.030278** |
| **Placebo** | **preD1** | **0.055868** | **0.054234** |
|  | **D2+7d** | **0.028387** | **0.036371** |
| **Placebo** | **preD1** | **0.013512** | **0.014788** |
|  | **D2+7d** | **0.014626** | **0.015933** |
| **Placebo** | **preD1** | **0.014204** | **0.027424** |
|  | **D2+7d** | **0.01518** | **0.009913** |
| **Placebo** | **preD1** | **0.022217** | **0.156138** |
|  | **D2+7d** | **0.011865** | **0.062038** |
| **Placebo** | **preD1** | **0.009187** | **0.008511** |
|  | **D2+7d** | **0.012094** | **0.005315** |
| **Placebo** | **preD1** | **0.075734** | **0.052487** |
|  | **D2+7d** | **0.08157** | **0.046084** |
| **Placebo** | **preD1** | **0.026073** | **0.0258** |
|  | **D2+7d** | **0.045697** | **0.027457** |
| **Placebo** | **preD1** | **0.011884** | **0.009035** |
|  | **D2+7d** | **0.017141** | **0.020794** |

*Non-stimulated refers to DMSO controls

References:

25. Kim, L. *et al.* Hospitalization Rates and Characteristics of Children Aged <18 Years Hospitalized with Laboratory-Confirmed COVID-19 - COVID-NET, 14 States, March 1-July 25, 2020. *MMWR Morb Mortal Wkly Rep* **69**, 1081-1088 (2020). <https://doi.org/10.15585/mmwr.mm6932e3>
